# Ternary Pt-Ru-Ni catalytic layers for methanol electrooxidation prepared by electrodeposition and galvanic replacement

**DOI:** 10.3389/fchem.2014.00029

**Published:** 2014-06-10

**Authors:** Athanasios Papaderakis, Nikolaos Pliatsikas, Chara Prochaska, Kalliopi M. Papazisi, Stella P. Balomenou, Dimitrios Tsiplakides, Panagiotis Patsalas, Sotiris Sotiropoulos

**Affiliations:** ^1^Physical Chemistry Laboratory, Department of Chemistry, Aristotle University of ThessalonikiThessaloniki, Greece; ^2^Centre for Research and Technology Hellas, Chemical Process and Energy Resources InstituteThessaloniki, Greece; ^3^Department of Physics, Aristotle University of ThessalonikiThessaloniki, Greece

**Keywords:** electrocatalysis, platinum catalysts, galvanic replacement, ternary catalysts, methanol oxidation

## Abstract

Ternary Pt-Ru-Ni deposits on glassy carbon substrates, Pt-Ru(Ni)/GC, have been formed by initial electrodeposition of Ni layers onto glassy carbon electrodes, followed by their partial exchange for Pt and Ru, upon their immersion into equimolar solutions containing complex ions of the precious metals. The overall morphology and composition of the deposits has been studied by SEM microscopy and EDS spectroscopy. Continuous but nodular films have been confirmed, with a Pt ÷ Ru ÷ Ni % bulk atomic composition ratio of 37 ÷ 12 ÷ 51 (and for binary Pt-Ni control systems of 47 ÷ 53). Fine topographical details as well as film thickness have been directly recorded using AFM microscopy. The composition of the outer layers as well as the interactions of the three metals present have been studied by XPS spectroscopy and a Pt ÷ Ru ÷ Ni % surface atomic composition ratio of 61 ÷ 12 ÷ 27 (and for binary Pt-Ni control systems of 85 ÷ 15) has been found, indicating the enrichment of the outer layers in Pt; a shift of the Pt binding energy peaks to higher values was only observed in the presence of Ru and points to an electronic effect of Ru on Pt. The surface electrochemistry of the thus prepared Pt-Ru(Ni)/GC and Pt(Ni)/GC electrodes in deaerated acid solutions (studied by cyclic voltammetry) proves the existence of a shell consisting exclusively of Pt-Ru or Pt. The activity of the Pt-Ru(Ni) deposits toward methanol oxidation (studied by slow potential sweep voltammetry) is higher from that of the Pt(Ni) deposit and of pure Pt; this enhancement is attributed both to the well-known Ru synergistic effect due to the presence of its oxides but also (based on the XPS findings) to a modification effect of Pt electronic properties.

## Introduction

Electrochemistry offers a clean and efficient route for energy production and storage: the electron is a clean reactant/product and electrochemical cells do not suffer from the Carnot cycle limitations of thermal engines (Denno, 1988; Narayan and Viswanathan, 1998; Chouhan and Liu, 2012). Among the main electrochemical energy conversion systems (fuel cells, solar cells, and batteries), fuel cells are characterized by high energy density and speed of recharging-refueling (Srinivasan, 2006; Viswanathan and Scibioh, 2007; Kreuer, 2013). The oxidant in a fuel cell is almost always oxygen (in few systems, hydrogen peroxide can act as such too) while the most common fuel in low temperature applications is hydrogen gas. For microfuel cell applications however (i.e., portable devices such as music players, laptops etc) liquid fluids are being explored, with methanol, borohydride, and formic acid being the best candidates (Yeom et al., 2005; La O' et al., 2007; Kamarudin et al., 2009). Direct methanol fuel cells (DMFCs) have a number of advantages, including a large theoretical energy density, a low operating temperature and the simplicity of storing, distributing, and handling a liquid chemical (Lamy et al., 2000; Hamnett, 2003). The main parameters that limit their performance and widespread establishment are methanol crossover to the cathode (whereby its spontaneous oxidation lowers the cathode potential) and anode poisoning by the adsorption of CO formed as an intermediate (Antolini et al., 2008; Yong et al., 2009; Brouzgou et al., 2012; Ehteshami and Chan, 2013; Tiwari et al., 2013). The first problem can be tackled by the development of improved membrane separators between the anode and cathode departments (Yong et al., 2009; Brouzgou et al., 2012) as well as the search for methanol-tolerant cathodes (Antolini et al., 2008); the second problem has prompted decades of electrochemical research (both theoretical and experimental) into modified Pt-based catalysts that are CO-tolerant (Petry et al., 1965; Beden et al., 1981; Shibata and Motoo, 1986, 1987; Hamnett and Kennedy, 1988; Ishikawa et al., 2004; Demirci, 2007). Both binary (Page et al., 2000; Park et al., 2002; Zhang et al., 2004; Koffi et al., 2005; Martz et al., 2005; Salgado et al., 2005, 2006; Yang et al., 2005; Antolini et al., 2006a; Royochowdhury et al., 2006; Stassi et al., 2006; Yuan et al., 2006; Kristian and Wang, 2008) and ternary (Lima et al., 2001; Lamy et al., 2002; Cooper and McGinn, 2006; Liu et al., 2006; Wang et al., 2006; Antolini, 2007; Zhang et al., 2007; Huang et al., 2009; Kim et al., 2012; Poh et al., 2012; Zhang and McGinn, 2012; Lee et al., 2013; Li et al., 2013; Tusi et al., 2013) Pt-based systems have been tested, with Ru being accepted almost universally as the second metal, mainly due to its ability to form surface oxides and hydroxides that can oxidize and remove the CO intermediate. As far as the third metal is concerned, a variety of other candidates have been tested (usually transition metals) (Lima et al., 2001; Lamy et al., 2002; Cooper and McGinn, 2006; Antolini, 2007; Zhang and McGinn, 2012).

The most common preparation method for fuel cell catalysts is the impregnation/reduction method, whereby the support (high surface area carbons) is dispersed in a solution containing the metal catalyst in ionic form which, following its adsorption on the support, is reduced by gaseous hydrogen at high temperature or by dissolved agents in solution (Antolini, 2003, 2007; Rao and Trivedi, 2005). In another family of methods, the metal particles are formed first in a colloidal form by chemical reduction at moderate temperatures [using organic stabilizers such as in the Bonnemann or microemulsion procedures (Boutonnet et al., 1982; Bonnemann et al., 1991)] and then adsorbed on the high surface area carbon supports.

Since 2001 an alternative method for the preparation of poly-metallic catalyst layers (known as galvanic replacement or transmetalation) has been proposed (Brankovic et al., 2001a,b,c; Kokkinidis et al., 2001). It comprises of the spontaneous deposition of a noble metal from its ionic solutions onto a less noble metallic layer or substrate which in turn dissolves as an ionic species. The early variant of the method involved the complete replacement of a Cu underpotentially deposited monolayer on a metallic substrate (Brankovic et al., 2001a,b,c), while later ones expanded to the partial replacement of Pb and Cu electrodeposited polylayers (Kokkinidis et al., 2001; Van Brussel et al., 2002, 2003; Papadimitriou et al., 2007; Tegou et al., 2007), the partial replacement of other transition metals (e.g., Fe, Co, Ni) (Papadimitriou et al., 2008, 2010; Tegou et al., 2008, 2009, 2010, 2011) and the preparation of practical catalysts by the use of high area carbon supports as the substrate (where the less noble precursor metal was chemically deposited and the more noble metal (Pt) was subsequently anchored) (Mintsouli et al., 2013a,b). By its very nature, the method tends to produce structures of a noble-metal (M)-rich shell and of a less-noble-metal (M^/^)-rich core (denoted as M(M^/^) hereafter). This structure has the practical advantage of minimizing the use of the expensive noble metal (since it will be restricted to the surface) and the fundamental virtue of allowing the study of electronic only effects of the less noble metal on the noble metal catalytic activity since, as the former resides in the core of the system, it will not be in contact with the reaction medium but only with the noble metal over-layers.

Despite some papers on using transmetalation to deposit binary precious metal systems from mixed solutions of their ions (e.g., Pt-Au, Pt-Pd etc), only a couple of papers using ternary Pt-Ru(Co) (Zhao et al., 2008) and Pt-Ru(Pb) (Ando et al., 2009) catalysts for methanol oxidation have appeared. At the same time, although Pt-Ru-Ni ternary systems have also been tried in methanol oxidation (Liu et al., 2006; Wang et al., 2006; Zhang et al., 2007; Lee et al., 2013; Li et al., 2013; Tusi et al., 2013), these were prepared by co-reduction methods. The feasibility of preparing Ru(Ni) layers has also been proven, but the material was tested in the hydrogen evolution reaction (Bianchi et al., 2005). Thus, there is scope for investigating the possibility of preparing Pt-Ru-Ni ternary systems of the Pt-Ru(Ni) type, by the partial galvanic replacement of pre-formed Ni layers by Pt and Ru, upon the contact of the former with ionic solutions of the latter. In line with the strategy followed in our group, before departing on the development of practical Pt(M) catalysts supported on high surface area carbons (where M is chemically deposited), we investigate the replacement of M layers electrodeposited on flat glassy carbon substrates.

The aim of this work has been to prepare Pt-Ru(Ni) coatings by the surface transmetalation of electrodeposited Ni layers on glassy carbon (GC) supports and correlate their structure with their electrocatalytic activity toward the oxidation of methanol. In that direction, specific objectives of this research have been: (i) The microscopic (SEM, AFM) and spectroscopic (EDS, XPS) characterization of the catalysts to obtain information about the transmetalation mechanism and the interactions of the catalyst components; (ii) The characterization of the catalyst electroactive surface area by electrochemical (voltammetric) experiments in pure acidic solutions; (iii) The evaluation of the catalyst activity in the electrooxidation of methanol (by means of current vs. applied potential curves) and its correlation to its properties.

## Experimental

### Preparation of nickel and platinized nickel layers on glassy carbon

Ni has been electrodeposited on a glassy carbon, GC, disc electrode at −1.10 V vs. a Saturated Calomel Electrode (SCE), from a 0.01 M Ni sulfamate + 0.227 mM NiCl_2_ + 0.025 M H_3_BO_3_ deaerated solution. The cathodic charge passed during the constant potential electrolysis was controlled to be 380 mC cm^−2^, resulting in an even deposit [see SEM pictures in Papadimitriou et al. (2008, 2010)] of a ca 130 nm thickness [based on Faraday's law, assuming a 100% Ni deposition current efficiency and taking into account the density of Ni (Callister et al., 1997)].

The Ni-coated GC electrodes, Ni/GC, thus prepared were subsequently immersed for 30 min in either a 0.1 M HCl + 5 × 10^−5^ M K_2_PtCl_6_ solution or a 0.1 M HCl + 2.5 × 10^−5^ M RuCl_3_ + 2.5 × 10^−5^ M K_2_PtCl_6_ mixed solution. As previously reported (Bianchi et al., 2005), RuCl_3_ stock solutions were aged for a week before they were used. Spontaneous Pt and/or Ru deposition by partial Ni replacement may then occur according to the following transmetalation reactions:

(1) $$2\text{Ni/GC}+{\text{PtCl}}_{6}^{2-}\to \text{Pt/GC}+2{\text{Ni}}^{2+}+6{\text{Cl}}^{-}$$

(2) $$3\text{Ni/GC}+{\text{2RuCl}}_{3}\to \text{2Ru/GC}+3{\text{Ni}}^{2+}+6{\text{Cl}}^{-}$$

During this treatment the solution was continuously bubbled with purified N_2_, since the complete absence of O_2_ proved to be crucial toward the co-deposition of Pt and Ru particles onto the Ni surface (Bianchi et al., 2005). This is because oxygen reduction, having a high standard potential, is highly competitive with Pt and Ru deposition for coupling with Ni dissolution. Note that at the higher concentrations of Pt complexes used in cases that a simple bi-metallic Pt(Ni) catalyst was prepared (Mintsouli et al., 2013b), there was no need for oxygen exclusion since, once the latter is consumed, the cathodic reaction supporting Ni dissolution becomes exclusively that of Pt deposition. (See also Discussion at the end of Microscopic and spectroscopic characterization of Pt(Ni) and Pt-Ru(Ni) deposits on GC as to why low Pt and Ru concentrations have been used in this work).

### Microscopic and spectroscopic characterization of coatings

Scanning Electron Microscopy (SEM) micrographs were acquired with a JEOL 6300 microscope and the composition of the electrode coatings was determined by the companion Oxford ISIS 2000 X-ray, EDS (EDAX) facility.

True three-dimensional morphological features of the deposits as well their thickness were obtained with a NT-MDT Solver Pro Atomic Force Microscope, in the tapping (semi-contact) mode.

X-ray photoelectron spectroscopy (XPS) analysis of the catalyst surface layers was carried out in an Axis Ultra DLD system by Kratos Analytical using a monochromated Al-Ka1 X-ray beam as the excitation source. The analyzed area had an elliptical shape with the two axes being ~400 and 700 μm. The pass energy was 80 eV for survey scans and 20 eV for HR spectra; for the latter case, the pass energy resulted in a broadening (FWHM) of less than 500 meV for the Ag-3d line. The studied surfaces were cleaned of adventitious Carbon and other surface contaminants by using a 4 kV Ar^+^ ion beam for 2 min. Data interpretation was performed with the Kratos-Vision software.

### Electrochemical characterization of coatings

Linear and cyclic voltammetry (continuous current monitoring as the potential is varied linearly with time, either to one direction or via a periodic change of direction between two limits) was performed with the Autolab 100 system (EcoChemie, now Metrohm), using a three-compartment cell. The working electrode (Pt, Pt(Ni)/GC or Pt-Ru(Ni)/GC) was placed in the central chamber above a special capillary (luggin capillary) to which the second chamber, containing the Saturated Calomel Electrode (SCE) reference electrode ended. The tip was positioned underneath the center of the disc and at a constant distance of 2 mm (this was to ensure minimal and reproducible uncompensated ohmic losses, as set by established electrochemical practice whose foundations can be found in Newman (1996). The uncompensated resistance, measured by both the current-interrupt and positive feedback methods, translated to ohmic losses always lower than 2 mV. The third chamber contained a Pt coil that served as the counter electrode, placed in a compartment separated from that of the working electrode by a glass frit. To ensure minimum contamination, each different set of experiments was carried out in dedicated cells. Hence, different cells were used for the initial Ni deposition, the electrode activation-cleaning in acid, the study of surface electrochemistry and that of methanol oxidation.

Following Ni deposition and immersion into the platinum or platinum-ruthenium exchange solutions, the as prepared Pt(Ni)/GC or Pt-Ru(Ni)/GC electrodes were scanned repeatedly (typically, more than 60 times) at 1 V s^−1^ in a deaerated 0.1 M HClO_4_ solution between −0.30 and +0.60 V vs. SCE, ensuring that any unreacted surface Ni was anodically dissolved or passivated during exposure to positive potentials. The electrodes were then scanned again at 500 mV s^−1^ in a fresh deaerated 0.1 M HClO_4_ solution until a steady state voltammogram, typical of Pt or Pt-Ru surface electrochemistry, was recorded (typically, the voltammetric picture stabilized after less than 10 cycles). As an independent determination of their electroactive surface area and also as an evaluation of their catalytic activity toward CO oxidation, the electrodes were immersed in 0.1 M HClO_4_ saturated with CO (>99.99%; Air Liquide), held at +0.20 V vs. SCE for 5 min (for adsorption of CO to take place) and then, after dissolved CO had been removed by 10 min of N_2_ purging, scanned to more positive potentials at 500 mV s^−1^ so that the CO monolayer was oxidized. For the study of methanol oxidation, the electrodes were transferred to a 0.1 M HClO_4_ + 0.5 M MeOH deaerated solution where linear potential sweep voltammetry was carried out at 5 mV s^−1^, between +0.10 V and +0.60 V vs. SCE for three times so that a stabilized picture was obtained.

### Electrode materials and chemicals

These were similar to those of (Papadimitriou et al., 2010). Glassy carbon from Alfa Aesar (1 mm thick) was cut into 5 mm diameter discs and sealed into glass tubes with epoxy resin glue. Electrodes to be used for SEM/EDS and XPS experiments were prepared by connecting the carbon disc (Alfa Aesar 1 mm, cut into 3 mm diameter specimens) to a glass tube via a bridge made of a shrinkable thermoplastic tube. The latter was filled with mercury to ensure electrical contact between the disc and a commercial wire inserted from the open end of the glass tube.

Ni sulfamate (p.a. 99%) from Fluka, NiCl_2_ (puriss> 97%) from Merck and H_3_BO_3_ (puriss 98%)from Aldrich were used in the preparation of Ni deposition solutions. H_2_PtCl_6_ hexahydrate from Sigma-Aldrich (ACS reagent, ≥37.50% as Pt) was employed for the Pt exchange solution. RuCl_3_ hydrate was from Merck. HClO4 from Riedel, (puriss p.a., ACS reagent, ≥70%) was used in the working solutions and MeOH was also from Riedel (Chromasolv®, for HPLC, gradient grade, ≥99.9%).

## Results and discussion

### Microscopic and spectroscopic characterization of pt(Ni) and Pt-Ru(Ni) deposits on GC

**Figure 1:**
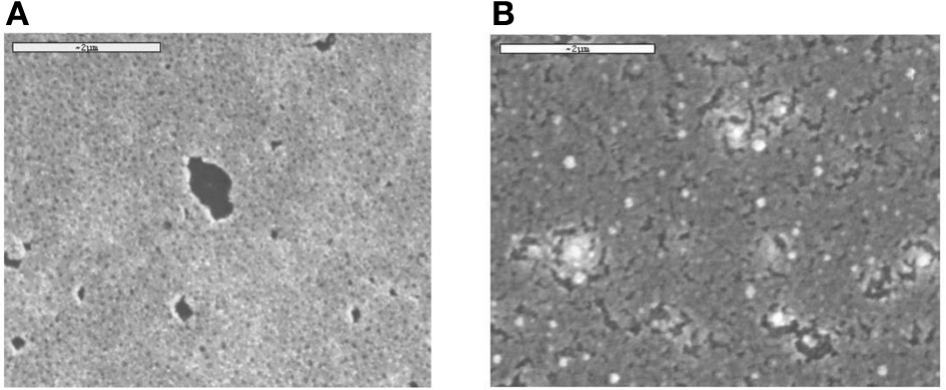
**SEM micrographs of: (A)** A Pt(Ni) coating of a 47% Pt-53%Ni atom composition, prepared after the immersion of Ni electrodeposits on GC (deposition charge density of 380 mC cm^−2^) in a 0.1 M HCl + 5 × 10^−5^ M K_2_PtCl_6_ solution, followed by electrochemical conditioning in the −0.3 ± 0.6 V vs. SCE potential range; **(B)** A Pt-Ru(Ni) coating of a 37% Pt-12% Ru-51%Ni atom composition, prepared from Ni electrodeposits [same as those of **(A)**] after their immersion in a 0.1 M HCl + 2.5 × 10^−5^ M RuCl_3_ + 2.5 × 10^−5^ M K_2_PtCl_6_ mixed solution and activated in a manner similar to **(A)**. Scale bar: 2 μm.

Figure 1A shows the SEM micrograph of a Pt(Ni) coating on a glassy carbon (GC) substrate, prepared by the immersion of a Ni/GC electrodeposit into a 5 × 10^−5^ M chloroplatinic acid exchange solution, after it has been electrochemically activated by cycling its potential between the onset of the hydrogen evolution region (−0.30 V) and the onset of surface oxide formation (+0.60 V). The morphology of the coating is similar to that observed in Papadimitriou et al. (2008, 2010), i.e., there is overall a uniform coverage of the substrate, decorated by nanopores and a few uncovered large patches. The pores and the patches result from significant Ni dissolution, either via the transmetalation process (coupled with Pt deposition at nearby locations) or by Ni electrochemical dissolution (induced by the positive potential applied to the electrode during its activation). Figure 1B shows the SEM micrograph of a Pt-Ru(Ni) coating, prepared by the immersion of the Ni/GC electrodeposit into a mixed, equimolar (2.5 × 10^−5^ M) chloroplatinic acid + ruthenium chloride solution, following its electrochemical activation. A less homogeneous and rougher coating is obtained, with both the deposit and uncovered areas organized in irregular patches. EDS elemental analysis of the deposits gives a Pt ÷ Ru ÷ Ni % bulk atomic composition ratio of 37 ÷ 12 ÷ 51 (and 47 ÷ 53 for the binary Pt-Ni control system).

**Figure 2:**
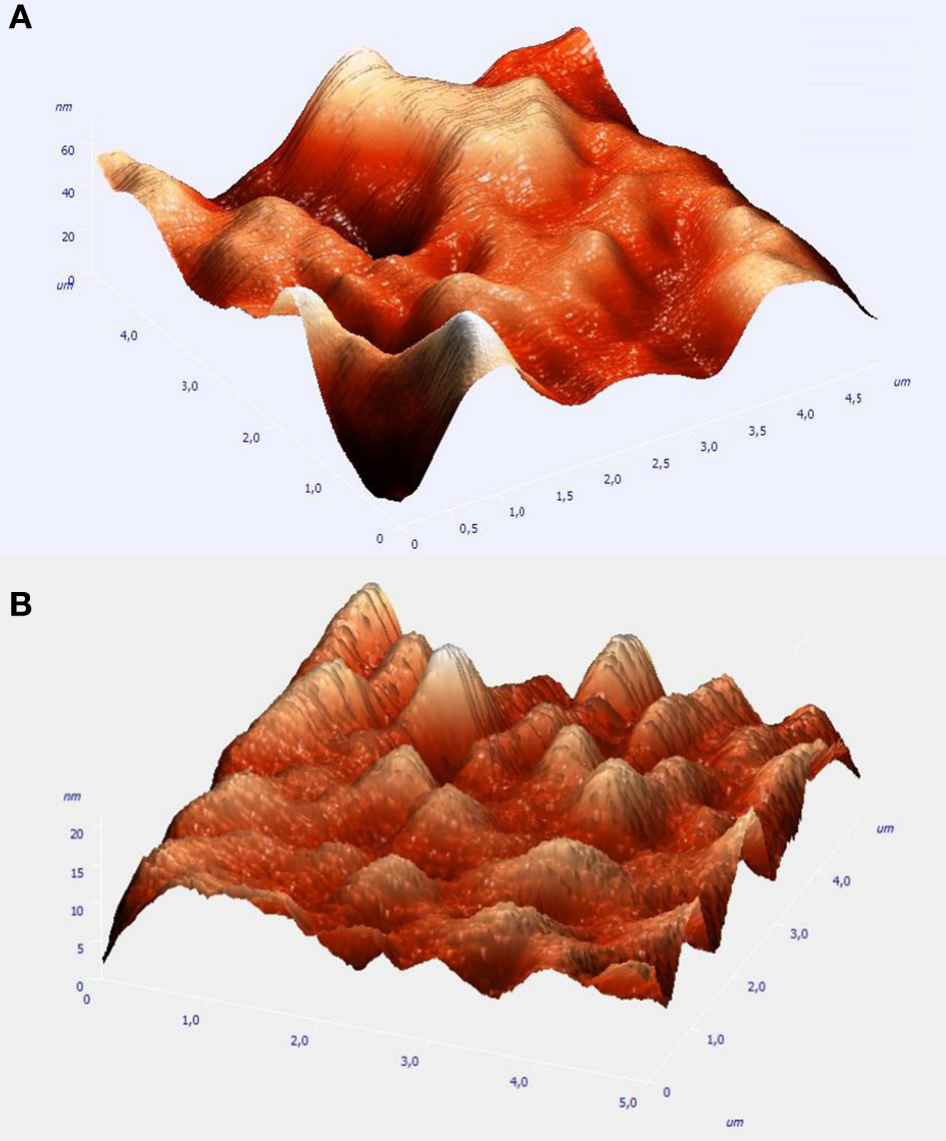
**AFM micrographs of 5 × 5 μm sample areas of: (A)** the Pt(Ni) coating of Figure 1A above; **(B)** the Pt-Ru(Ni) coating of Figure 1B above.

A direct estimate of the coating thickness is not possible by the SEM pictures in either case. It could be however inferred by the true topographical information supplied by AFM measurements. Figure 2 depict three dimensional representations of the low resolution AFM image taken over a 5 × 5 μ m area of the Pt(Ni) and Pt-Ru(Ni) deposits.

It can be seen that Pt(Ni) is organized in hundreds of nm to few μ m nodules, further aggregated in larger formations and separated by wells. Assuming that the deepest wells correspond to the GC substrate (darkest and largest areas of the corresponding SEM picture of Figure 1A), the maximum deposit height can be estimated to be in the 60–70 nm range. This is to be compared with the thickness of the initially electrodeposited smooth Ni layer, estimated as 130 nm (from the charge passed and Faraday's law, and by the density of bulk Ni-see also Experimental above). Both the perforated morphology and the thickness of the resulting Pt(Ni) layer point to a “thinning” process that occurs during the transmetalation step. At the low concentration levels of the Pt-containing solution of this study (so that Ru is also deposited-see discussion below), the rate of Ni replacement by Pt becomes slow due to mass transfer limitations (despite the relatively high standard potential difference of the two metals-see also below); hence the protection of the dissolving Ni from the outer Pt shell takes time before it is completed, allowing for significant Ni losses. In the case of Pt-Ru(Ni), the AFM picture of Figure 2B shows a deposit consisting of smaller nodules and of a maximum thickness in the range of 20–30 nm, indicating larger Ni losses during the transmetalation step. This is to be expected since in the mixed, equimolar solution, Pt is partially substituted by Ru which has a lower standard potential, hence lower kinetics of deposition; this is because not only the thermodynamics but also the kinetics of the transmetalation reactions (1) and (2) (see Experimental above) depend on the E^0^_PtCl_6_^−4^ /Pt_ − E^0^_Ni^2+^/Ni_ and E^0^_Ru(III)/Ru_ − E^0^_Ni^2+^/Ni_ differences (Papadimitriou et al., 2008). [Note that, according to (Lovrecek et al., 1985), E^0^_PtCl_6_^−4^/Pt_ = + 0.774 V vs. SHE, E^0^_Ru(III)Ru_ = +0.386 V vs. SHE, and E^0^_Ni^2+^/Ni_ = −0.257 V vs. SHE]. At this point we would like to stress that the reason of using solutions of low Pt concentration [in contrast to our previous studies (Papadimitriou et al., 2007, 2008, 2010; Tegou et al., 2007, 2008, 2009, 2010, 2011; Mintsouli et al., 2013a,b)] has been the deliberate introduction of mass transfer limitations so that the large difference in Pt and Ru deposition kinetics is attenuated and Ru co-deposition is accomplished. (Attempts to use higher Pt and Ru concentrations failed to produce mixed Pt-Ru deposits and instead, only Pt ones were obtained).

**Figure 3:**
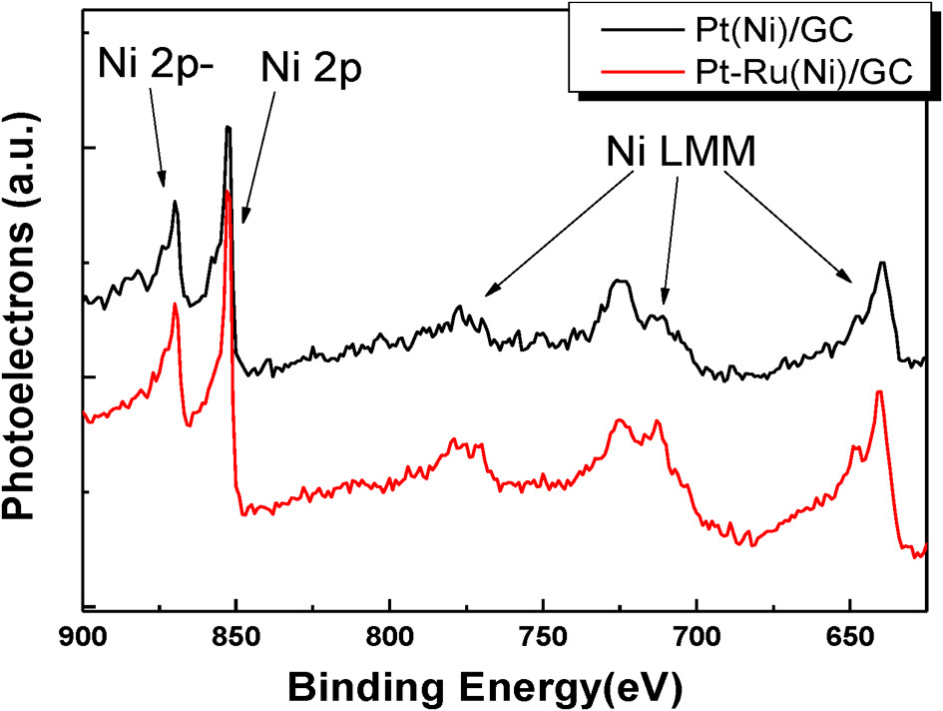
**XPS spectra in the Ni2p binding energy range for Pt(Ni) (upper curve) and Pt-Ru(Ni) (lower curve) coatings**.

In order to obtain further insight into the chemical state, the interactions and the composition of the outer layers of the deposits, XPS and X-Ray excited Auger Electron Spectroscopy (XAES) (two more surface sensitive techniques) were used. Figure 3 presents the combined XPS/XAES spectra for Ni.

It can be seen that the two main peaks observed in the 850–900 eV range are those corresponding to metallic Ni and no “satellite peaks” (attributed to Ni oxides and hydroxides Park et al., 2002; Mintsouli et al., 2013b) are pronounced in the binding energy region between these two peaks. This indicates that even in the few outer atomic layers that are probed by XPS, Ni is not present as an oxide, further hinting to its absence from the outmost surface layer (where, in contact with the solution, it would have been converted to oxides/hydroxides) and the formation of a complete, protective Pt or Pt-Ru shell.

**Figure 4:**
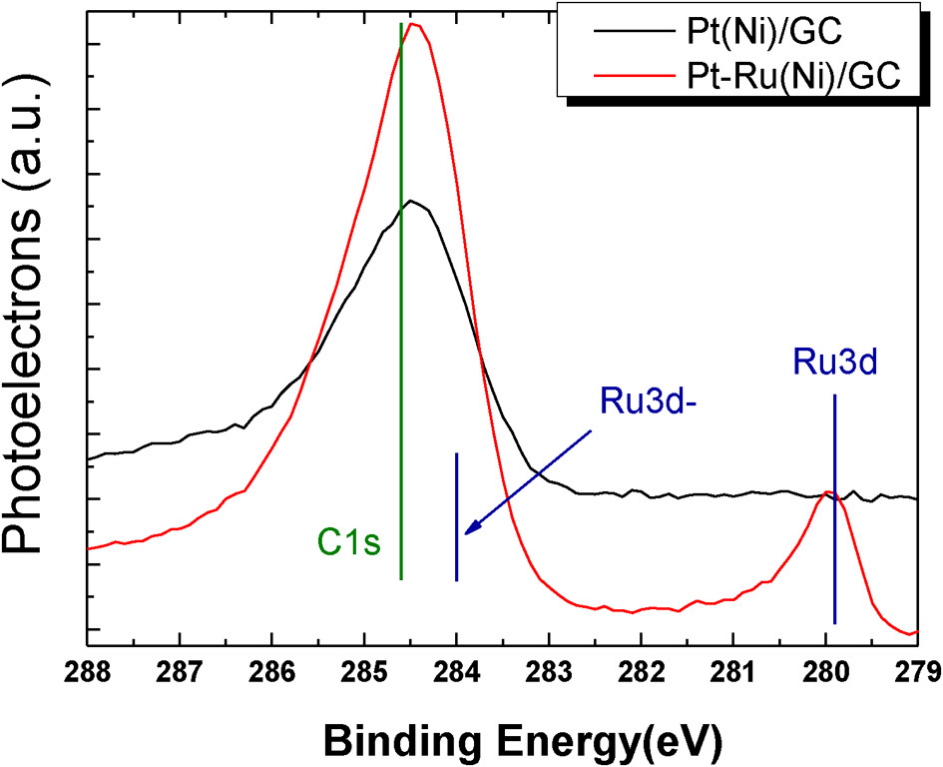
**XPS spectra in the C1s and Ru3d binding energy range for Pt(Ni) (upper curve) and Pt-Ru(Ni) coatings (lower curve)**.

Figure 4 shows the spectrum segment in the C1s and Ru 3d electron binding energies for the Pt(Ni) and Pt-Ru(Ni) catalysts respectively.

The C1s line corresponds to adventitious carbon, determined separately, set to the value of 284.8 eV reported in the literature (Jackson and Nuzzo, 1995) and used as a reference point for all the spectra recorded. The position of the C1s peak of Figure 4 is at 284.5 eV, as expected for the glassy carbon substrate (Leiro et al., 2003), accessible at uncovered areas. The peak around 280 eV observed in Figure 4 proves unequivocally the presence of Ru in the outer layers of the Pt-Ru(Ni) catalyst.

**Figure 5:**
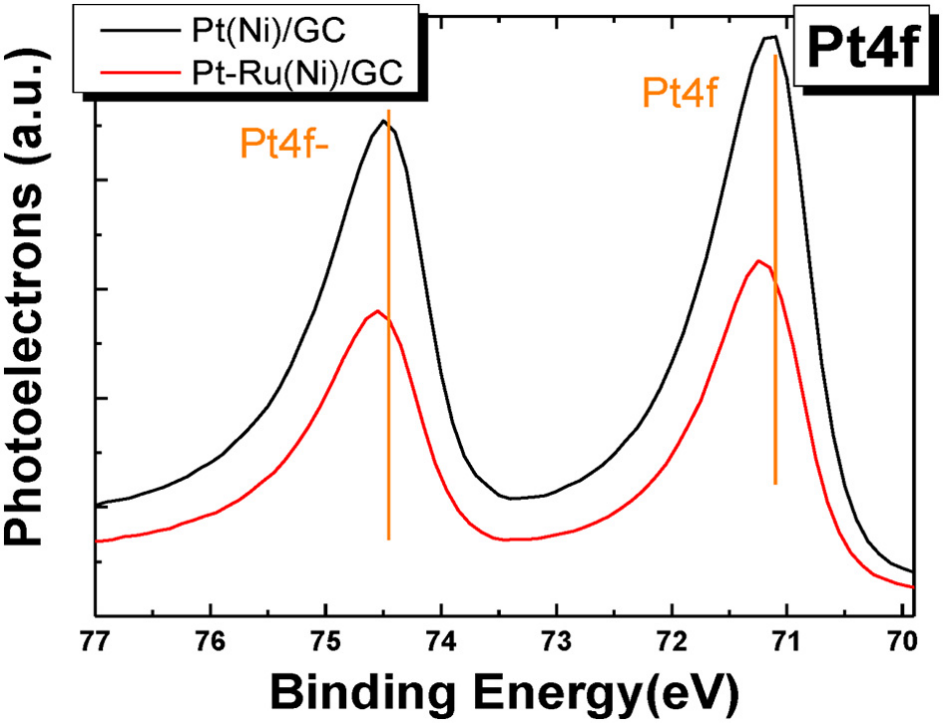
**XPS spectra in the Pt4f binding energy range for Pt(Ni) (upper curve) and Pt-Ru(Ni) (lower curve) coatings**.

Figure 5 presents the XPS spectra in the Pt 4f electron energy range for the Pt(Ni) and Pt-Ru(Ni) deposits.

In the former case, there is hardly any significant change in peak position (i.e., it is less than 0.1 eV), in accordance with our previous findings for similar Pt(Ni)/C systems (Mintsouli et al., 2013b), indicating weak interactions (undetected by XPS) of the Pt outer shell with the Ni-rich core. On the contrary, measurable 0.1 and 0.2 eV shifts to higher binding energy values (for the higher and lower binding energy Ptf peaks respectively) are observed in the case of the Pt-Ru(Ni) deposit (in accordance with extensive literature for Pt-Ru systems [see for example (Wakisaka et al., 2006; Zhou et al., 2007; Xu et al., 2010)], indicating strong Pt-Ru interactions. The origin of Pt electronic modification by smaller or less electronegative metal atoms is the strain experienced by Pt atoms residing on top of atoms with a smaller Wagner-Sweitz radius or partial charge transfer respectively. The theoretical work of Nørskov and co-workers (Ruban et al., 1997; Kitchin et al., 2004; Nørskov et al., 2004) specifies this modification as a downshift of the electron d-band center [confirmed by UPS measurements (Stamenkovic et al., 2006, 2007)], whereas Watanabe and co-workers further specify this as a lowering of core electron energy levels (confirmed by XPS measurements) (Wakisaka et al., 2006, 2008). Since Ni atoms are smaller and less electronegative than Ru, one would expect that Pt modification would be pronounced in the case of Pt(Ni) systems too. The fact that it is only observed for Pt-Ru(Ni) indicates that either the Pt and Pt-Ru shells are too thick for any effects of the underlying Ni to be operative at all or that the composition of the films under the shells is such that these effects are too weak to be measured by XPS (that could be due to the fact that the underlying Ni is intermixed with Pt or Pt and Ru and hence its modifying effect attenuated). Arguments against the existence of a very thick Pt shell can be found in Papadimitriou et al. (2010) where Auger electrons originating from Ni can be detected during AES experiments on similar Pt(Ni) samples as well as from the combined XPS/AES results of Figure 3. Based on this finding and the inelastic mean free path of the corresponding Auger electrons through Pt, it has been estimated in Papadimitriou et al. (2010) that the outer Pt shell could be thinner than the 4–5 layers to which, according to (Meier et al., 2004; Kibler, 2008), the strain effect of the under-layers can extend.

Evidence for the enrichment of the outer layers with Pt and Pt-Ru and their depletion from Ni is provided by XPS elemental analysis which gives a Pt ÷ Ni % surface atomic composition ratio of 85 ÷ 15 and a Pt ÷ Ru ÷ Ni ratio of 61 ÷ 12 ÷ 27, i.e., a clear Pt and Pt-Ru enrichment of the outer layers when compared to the bulk composition (obtained by EDS and quoted above as 47 ÷ 53 and 37 ÷ 12 ÷ 51 respectively). On the contrary, the co-existence of Ru in the outer layers of the deposit (a result of the galvanic replacement process itself) allows for its stronger interaction with Pt on the surface, reflected in a shift of its peaks.

### Electrochemical characterization of Pt(Ni)/GC and Pt-Ru(Ni)/GC electrodes

**Figure 6:**
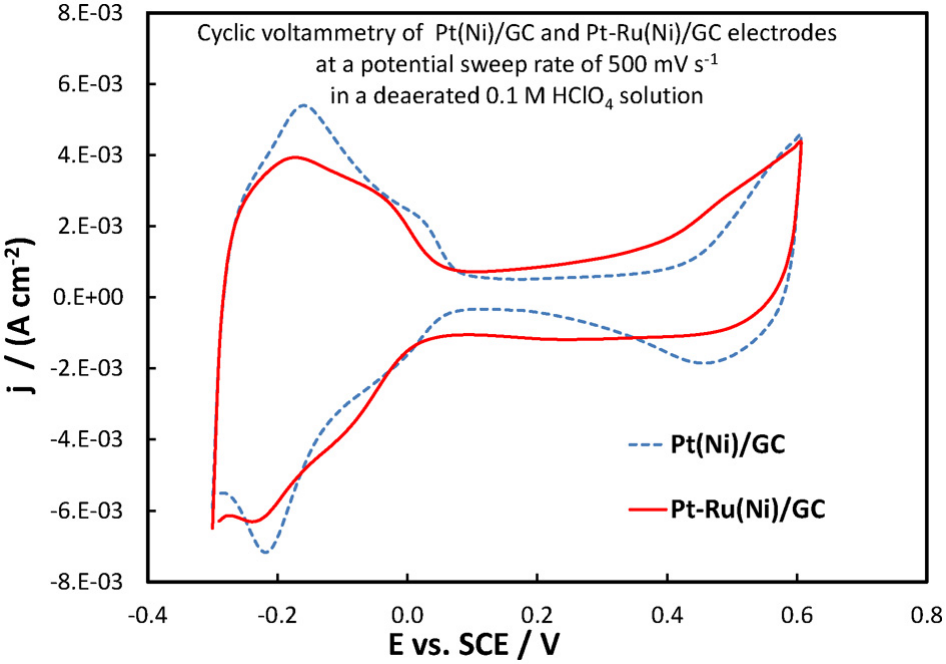
**Voltammograms of Pt(Ni)/GC and Pt-Ru(Ni)/GC electrodes (as indicated in the graph), in a deaerated 0.1 M HClO_4_ solution at 500 mV s^−1^ potential scan rate**.

**Figure 7:**
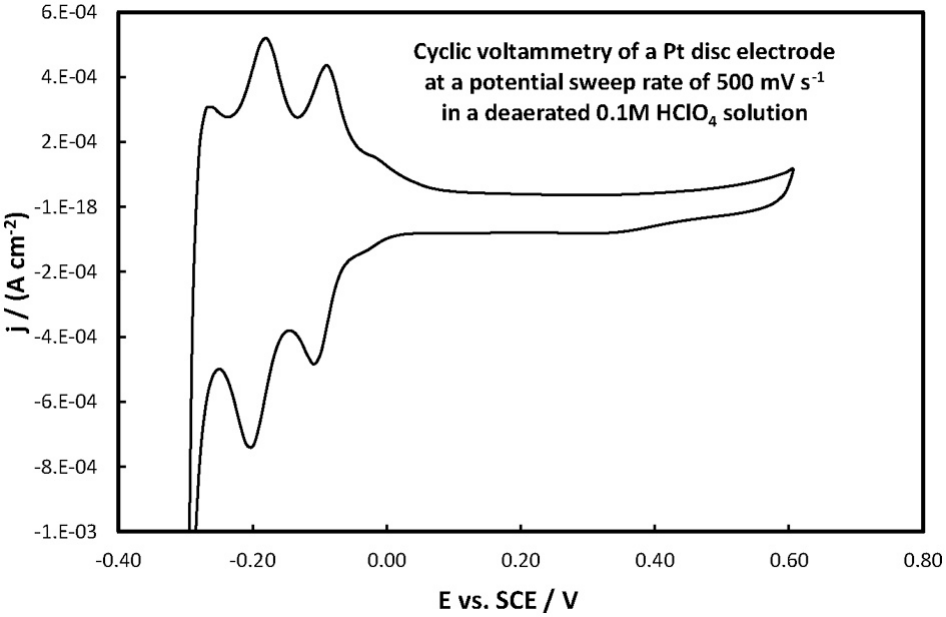
**Voltammogram of a Pt disc electrode in a deaerated 0.1 M HClO_4_ solution at 500 mV s^−1^ potential scan rate**.

The electrochemical properties of the catalysts' surface were probed by means of current vs. potential curves recorded during the periodic variation of the potential within a given potential range, i.e., by means of cyclic voltammetry. Figure 6 presents the cyclic voltammograms (obtained at fast potential sweep rates of 500 mV s^−1^) for the Pt(Ni)/GC and Pt-Ru(Ni)/GC electrodes in a deaerated 0.1 M HClO_4_ solution, whereas Figure 7 that for a Pt disk electrode.

**Figure 8:**
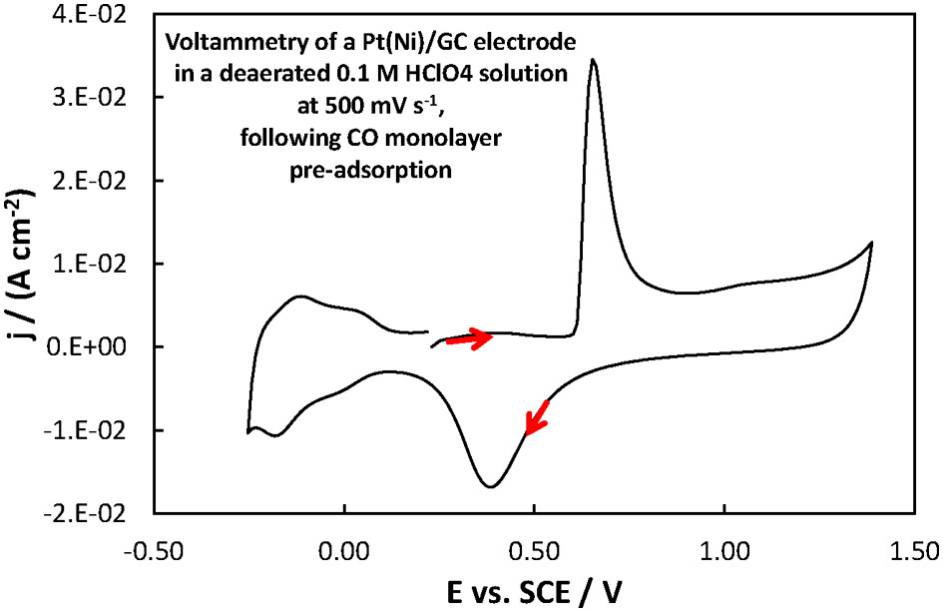
**Voltammogram of a Pt(Ni)/GC electrode in a deaerated 0.1 M HClO_4_ solution at 500 mV s^−1^, following the procedure for CO monolayer adsorption and subsequent oxidation described in section Electrochemical characterization of coatings**. The scan was initiated at +0.20 V toward more positive potentials.

For the Pt(Ni)/GC electrode features similar to those of pure polycrystalline Pt [(Tegou et al., 2007) and Figure 7] are seen. These comprise of the cathodic (negative current) peaks, attributed to hydrogen electrosorption on Pt as an atomic layer (at ca. 0.00 and −0.20 V) and their conjugate anodic (positive current) peaks, attributed to its oxidative desorption upon potential reversal. At positive potentials the anodic currents that start to increase at ca 0.50 V correspond to the onset of Pt surface oxide formation while the cathodic peak at the same potential corresponds to its reductive removal/stripping. The similarity of the voltammetric features with those of pure Pt is further evidence of the absence of any Ni from the catalyst surface (as also inferred from the Ni XPS spectrum discussed in the previous section). The slight difference between Pt(Ni) and Pt with respect to the relative heights of the strongly bound (at ca. 0.00 V) and weakly bound H (at ca. −0.20 V), was also observed in Tegou et al. (2009, 2010, 2011, Papadimitriou et al., 2010) and should be due to the effect of Ni on the adsorption strength of Pt (see also discussion for methanol oxidation below). Using the charge density associated to the formation or stripping of a H monolayer [210 μ C cm^−2^ (Tegou et al., 2007)] and the charge corresponding to H stripping from the catalyst (calculated from the area under the curve segment between the H desorption peaks, after subtraction of the capacitive current recorded in the double layer region, between 0.10 and 0.30 V, assuming that this remains constant during the desorption process), an estimate of the Pt electroactive surface area, A_e_, can be made. From that, the ratio *r* = A_e_/A_g_(known as roughness factor, with A_g_ being the substrate geometric area) can be estimated as 12.8 cm^2^ of Pt per 1 cm^2^ of substrate geometric area, in accordance with the micro-particulate/rough texture of the deposit, as shown in the SEM and AFM pictures above. At first sight, the use of the hydrogen adsorption/desorption charge and the value of 210 μ C cm^−2^, to find the electroactive area of Pt(Ni) electrodes, contradicts the findings of (Fowler et al., 2008) for PtNi(111) and of (Hoffmannová et al., 2013) for nanoparticulate PtNi alloys, in which the authors reported a H coverage less than one. However, those electrodes were different than the ones prepared for this work: those of (Fowler et al., 2008) had a monocrystalline underlayer with the one in contact with the Pt shell being a pure Ni layer; those of (Hoffmannová et al., 2013) were conditioned in the hydrogen adsorption region and had Ni surfacing to the outer shell too. An indication that the approach used to find the electroactive area is legitimate in the case of our Pt(Ni) electrodes is provided by the fact that a very similar electroactive area could be estimated by using the charge corresponding to the oxidation of a CO monolayer in the experiment depicted in Figure 8.

The roughness factor estimated by H desorption (taking the value of 210 μ C cm^−2^ for a complete H monolayer) was 13.9 whereas that based on CO oxidative 2e^−^ desorption (assuming 420 μ C cm^−2^ for a complete monolayer of linearly bonded CO) could be found as 13.1 (i.e., almost the same). If the effect of Ni on the adsorption properties of Pt in our samples was so strong as to affect the coverage of adsorbed H and CO it would be very unlikely that the extent of decrease in coverage would be identical for both species.

In the case of the Pt-Ru(Ni)/GC electrode, three features of the corresponding voltammogram of Figure 6 point to the co-existence of Ru at the catalyst surface (Gasteiger et al., 1993; Chu and Gilman, 1996; Tripkovic et al., 2002). First, the hydrogen adsorption/desorption peaks in the 0.00 to −0.30 V range are ill-defined, as expected if surface sites were also occupied by Ru, since that does not show significant H atom adsorption/desorption and the latter processes partially overlap with some Ru oxide reduction/formation. Second, the region of the voltammogram between 0.00 and +0.40 V (known as double layer region, where any current recorded in the case of pure Pt does not arise from a faradaic red/ox process but it is simply a capacitive, electrode charging current) is characterized by higher currents, indicative of Ru electrochemistry. Finally, the onset potential at which the formation of surface oxides occurs at the Pt-Ru(Ni) electrode (indicated by a current rise during the positive potential scan, beyond ca +0.40 V) is less positive than that for Pt(Ni). This could be explained by the presence of Ru on the surface whereby, in the presence of water, Ru surface oxides are formed easier/at lower potentials than Pt surface oxides. For the case of Pt-Ru(Ni)/GC too, the Pt electroactive area is similar to that of Pt(Ni)/GC. Since both EDS and XPS analysis confirmed that there was less Pt in the ternary catalyst, similar electroactive areas could only be explained if this decrease in Pt content is accompanied by an increase in the Pt-Ru(Ni) deposit roughness (as confirmed at microscopic level by the SEM pictures of Figure 1). It should be noted that the use of the charge under the hydrogen adsorption or desorption region in the case of PtRu electrodes will most likely lead to an overestimate of Pt electroactive area since it contains charge associated with Ru oxide processes too (Gasteiger et al., 1993); however if this overestimate is accounted for/corrected the electroactive area-specific activity of the electrode toward a Pt-catalyzed reaction (such as methanol oxidation) would further increase.

### Methanol and CO oxidation in acid

**Figure 9:**
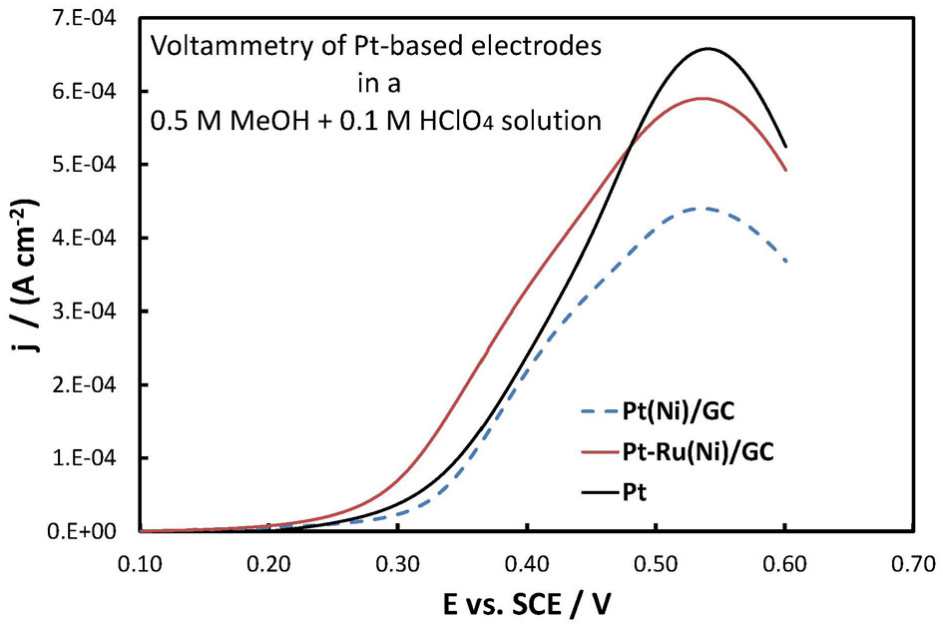
**Voltammograms (third positive-going scan at 5 mV s^−1^ potential scan rate) of Pt(Ni)/GC, Pt-Ru(Ni)/GC, and bulk Pt electrodes (as indicated in the graph) in a deaerated 0.5 MeOH + 0.1 M HClO_4_ solution**. Current density is per electroactive surface area.

The electrochemical oxidation of methanol at Pt-based electrodes was studied by near-steady state, slow linear potential sweep (at 5 mV s^−1^) voltammetry, in a 0.5 M MeOH + 0.1 M HClO_4_ solution. Figure 9 presents the corresponding voltammograms (positive-going potential sweeps) for Pt(Ni)/GC, Pt-Ru(Ni)/GC and pure Pt electrodes. The current density is reported per Pt electroactive area (as estimated from H monolayer adsorption-see above) so as to depict the inherent catalytic activity of the material and correct for mere increased surface area effects.

They all show the peak-shaped characteristics of methanol oxidation [(Papadimitriou et al., 2007, 2010) and references therein]. Up to the peak potential of ca. +0.55 V chemisorbed methanol and the intermediate/poison CO are oxidized at an increasing rate on Pt free metal sites (Pt is only partially covered by Pt oxides in that potential range). At more positive potentials the higher coverage of Pt by its surface oxides limits the number of free Pt centers necessary for the initial reaction step of methanol chemisorption, thus decreasing the observed current.

Both Pt-Ru(Ni) and, mainly, Pt(Ni) catalysts show smaller peak currents than plain Pt. According to the above-mentioned, generally accepted, interpretation of the shape of methanol oxidation voltammogram, this may arise from a higher Pt surface oxide coverage at these potentials, on Ni containing systems The surface voltammetry of Pt(Ni) and Pt(Co) electrodes presented in Tegou et al. (2010) and the experimental findings of high OH species coverage at Pt-Fe electrodes (Wakisaka et al., 2008), do indeed support this idea. In the case of Pt-Ru(Ni) the effect is less pronounced since the oxides are preferentially formed on Ru than on Pt. At this point we would like to stress the fact that electrochemical measurements, due to their surface specific nature, can sometimes provide macroscopic evidence for subtle interactions (in this case, Pt-Ni interactions) that even surface sensitive spectroscopic techniques (in this case XPS) fail to provide.

**Figure 10:**
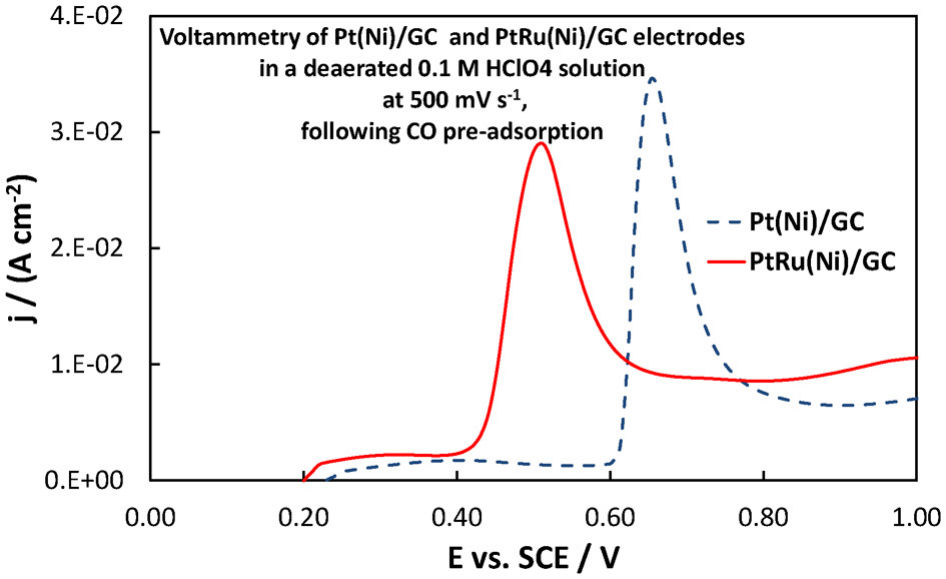
**Voltammograms of PtRu(Ni)/GC and Pt(Ni) electrodes in a deaerated 0.1 M HClO_4_ solution at 500 mV s^−1^, following the procedure for CO monolayer adsorption and subsequent oxidation described in section Electrochemical characterization of coatings**. The scan initiated at +0.20 V toward more positive potentials.

**Figure 11:**
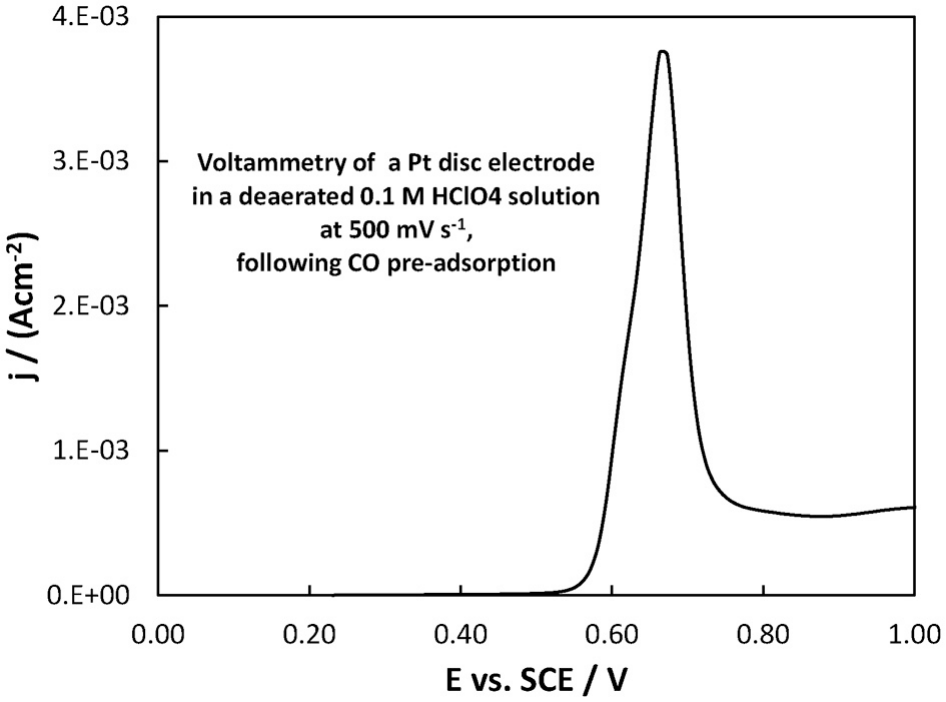
**Voltammogram of a Pt disc electrode in a deaerated 0.1 M HClO_4_ solution at 500 mV s^−1^, following the procedure for CO monolayer adsorption and subsequent oxidation described in section Electrochemical characterization of coatings**. The scan initiated at +0.20 V toward more positive potentials.

From a fuel cell catalyst point of view, the potential range of interest for methanol oxidation is at low ovepotentials, i.e., in the potential range below +0.40 V vs. SCE. In that range, the superiority of the Pt-Ru(Ni) catalyst compared to the Pt(Ni) and plain Pt is clearly seen. This is in complete accordance with the well-established view (Gasteiger et al., 1993; Chu and Gilman, 1996; Tripkovic et al., 2002) that the presence of Ru on the surface of a Pt-based catalyst is beneficial for methanol oxidation since its oxides accelerate CO oxidative removal from nearby Pt sites, at low overpotentials where formation of Pt oxides is limited and where the applied potential is not high enough for direct CO electro-oxidation. This can be supported by the voltammograms of Figures 10, 11 showing the oxidation of adsorbed CO at the three electrodes studied (recorded at a scan rate of 500 mV s^−1^), whereby CO is oxidized at less positive potentials on the PtRu(Ni) electrode.

On the other hand, the near-coincidence of the Pt(Ni) and Pt curves for methanol oxidation (Figure 9) is in line with results reported in the literature for Pt-Ni and Pt-Co carbon supported catalysts (Yang et al., 2005; Antolini et al., 2006b) as well as with our own recent voltammetric results on Pt(Ni) electrodes prepared by transmetalation (Papadimitriou et al., 2010). The inefficiency of Pt(Ni) catalysts (at least in the short period timescale of voltammetric experiments) has been interpreted by the fact that the lowering of the d-band center energy, ε_d_, of Pt and the associated decrease in its adsorption affinity for small atoms and molecules (Ruban et al., 1997; Kitchin et al., 2004; Nørskov et al., 2004), results in an interplay between decreased methanol chemisorption (decreasing the overall rate of oxidation) and decreased CO intermediate poison adsorption (increasing the rate of oxidation).

## Conclusions

Ternary Pt-Ru-Ni catalyst layers have been prepared on a glassy carbon, GC, substrate by galvanic partial replacement of electrodeposited Ni layers, upon their immersion at room temperature in mixed Pt and Ru salt solutions.

Both the surface electrochemistry of the ternary catalyst as well as its XPS spectra point to the existence of a Pt-Ru shell and the recession of Ni in the interior of the layers.

The ternary catalyst exhibited high electrocatalytic activity toward methanol electrooxidation at low overpotentials, highlighting the further opportunities offered by the method for the preparation of efficient practical Pt-Ru catalysts on high surface area carbon supports for use in direct methanol fuel cells.

### Conflict of interest statement

The authors declare that the research was conducted in the absence of any commercial or financial relationships that could be construed as a potential conflict of interest.
